# Whole-Genome Sequencing Analysis of Non-Typhoidal *Salmonella* Isolated from Breeder Poultry Farm Sources in China, 2020–2021

**DOI:** 10.3390/antibiotics12111642

**Published:** 2023-11-19

**Authors:** Zijing Ju, Lulu Cui, Changwei Lei, Mengze Song, Xuan Chen, Ziwei Liao, Tiejun Zhang, Hongning Wang

**Affiliations:** 1 Laboratory of Bio-Resource and Eco-Environment, Ministry of Education, College of Life Sciences, Sichuan University, Chengdu 610017, China; juzijing@stu.scu.edu.cn (Z.J.); leichangwei@scu.edu.cn (C.L.); 2019222040101@stu.scu.edu.cn (X.C.); liaoziweivv@stu.scu.edu.cn (Z.L.); 2021222040041@stu.scu.edu.cn (T.Z.); 2 Animal Disease Prevention and Food Safety Key Laboratory of Sichuan Province, Chengdu 610064, China; 3 Shandong Key Laboratory of Animal Biotechnology and Disease Control and Prevention, College of Animal Science and Veterinary Medicine, Shandong Agricultural University, Tai’an 271018, China; 2020010090@sdau.edu.cn (L.C.); songmz@sdau.edu.cn (M.S.)

**Keywords:** non-typhoidal *Salmonella*, public health, breeder chicken, whole-genome sequencing, antimicrobial resistance, virulence

## Abstract

Non-typhoidal salmonellosis is a dangerous foodborne disease that causes enormous economic loss and threatens public health worldwide. The consumption of food, especially poultry or poultry products, contaminated with non-typhoidal *Salmonella* (NTS) is the main cause of human salmonellosis. To date, no research has identified the molecular epidemiological characteristics of NTS strains isolated from breeder chicken farms in different provinces of China. In our study, we investigated the antimicrobial resistance, phylogenetic relationships, presence of antimicrobial resistance and virulence genes, and plasmids of NTS isolates recovered from breeder chicken farms in five provinces of China between 2020 and 2021 by using a whole-genome sequencing (WGS) approach and phenotypic methods. All sequenced isolates belonged to six serovars with seven sequence types. Nearly half of the isolates (44.87%) showed phenotypic resistance to at least three classes of antimicrobials. *Salmonella enterica* serotype Kentucky harbored more antimicrobial resistance genes than the others, which was highly consistent with phenotypic resistance. Furthermore, the carried rate of 104 out of 135 detected virulence genes was 100%. Overall, our WGS results highlight the need for the continuous monitoring of, and additional studies on, the antimicrobial resistance of NTS.

## 1. Introduction

Foodborne diseases caused by *Salmonella* present a significant public health concern around the world [1]. *Salmonella* (*S*.) *enterica* subsp. *enterica* is generally classified into typhoidal *Salmonella* and non-typhoidal *Salmonella* (NTS) [2]. NTS serotypes are characterized by a broader host range than typhoidal *Salmonella* and usually present self-limiting gastroenteritis. Worldwide, NTS is among the primary causes of human foodborne diseases and outbreaks [3,4] and is responsible for an estimated 93.8 million cases of gastroenteritis and approximately 155,000 deaths per year [5]. Contaminated poultry and poultry products are considered to be the main sources of NTS infections in humans [6,7].

Investigating clonal relationships and genomic diversity among *Salmonella* strains is now a vital task. According to the Kauffmann–White–Le Minor scheme, as of 2019, more than 2600 serotypes of *Salmonellae* have been identified, 1600 of which belong to *S. enterica* subsp. *enterica* [8]. To date, more than 200 *Salmonella* serovars have been confirmed as causing human salmonellosis [9]. It is reported that *S*. Enteritidis and *S*. Typhimurium remain the top two serotypes that result in clinical cases of salmonellosis [10]. Moreover, *S*. Thompson has been confirmed as the principal cause of several outbreaks of foodborne diseases [11,12,13,14].

Although traditional antigen-based serotyping continues to be frequently used to identify *Salmonella*, this is a time-consuming means of differentiating similar isolates within the same serovar [15]. As an alternative, whole-genome sequencing (WGS) has become widely applied in epidemiological investigations and the public health surveillance of *Salmonella* [15,16].

The emergence and transmission of antimicrobial resistance (AMR) in NTS have presented an increasingly serious public health threat in both developed and developing countries [17,18,19,20]. Antimicrobials widely used to treat salmonellosis in veterinary and human medicine exert selective pressure on resistant strains [21,22]. Of particular concern, increasing numbers of multidrug-resistant (MDR) NTS, defined as having acquired non-susceptibility to at least one agent in three or more antimicrobial categories [23], have been reported in recent years [9]. MDR strains, which can be transmitted to humans through contaminated poultry and poultry products, pose a serious threat to public health [24]. Consequently, it is necessary to continuously monitor the prevalence and AMR of NTS in different geographical areas. Research has shown that AMR is achieved via the horizontal transmission of AMR genes (ARGs) and chromosomal mutations [25]. Furthermore, *Salmonella* strains acquire ARGs largely through the acquisition of plasmids [26]. Thus, as a convenient and rapid technology for AMR analysis, WGS can be used to identify the presence of resistance genes and plasmids along with the possible genetic determinants responsible for the mechanisms of AMR [18,27].

The pathogenesis of *Salmonella* relies on the concerted efforts of multiple virulence factors [28], which are necessary for *Salmonella* to adhere to, invade, and replicate inside host cells. The relevant virulence genes are primarily located on the chromosome, *Salmonella* pathogenicity islands (SPIs), virulence plasmids, and bacteriophages. Of these, SPIs are chromosomal regions that encode different virulence genes [29]. Thus far, more than 20 SPIs have been identified, each of which plays a different role in the infection and prevalence of different serovars [30,31]. Numerous studies have investigated the genetics of *Salmonella*’s pathogenicity; even so, there are many virulence genes whose functions have yet to be studied [28,32]. To that end, using WGS technology can be beneficial for estimating the potential pathogenicity of *Salmonella*.

China is considered to be a large producer and consumer of poultry. Previous studies have described the characteristics and AMR of *Salmonella* strains isolated in China [33,34]; however, the molecular epidemiological characteristics of NTS strains isolated from breeder chicken farms in different provinces of China have yet to be examined. Therefore, the objective of our study was to investigate AMR and the presence of virulence factors in NTS recovered from breeder chicken farms in different provinces of China while also analyzing the genetic relationships between different farms. In our study, multiple strains were isolated from the same farms in different years so that we could longitudinally compare the characteristics of the temporal variation in strains isolated from each farm. To our knowledge, our study is the first to use a WGS approach to investigate the molecular epidemiological characteristics of NTS strains isolated from breeder chicken farms in five provinces of China. Our findings add valuable information to the epidemiology of NTS in China, where the emergency of MDR *Salmonella* continues to evolve.

## 2. Results

### 2.1. Distribution of Serotypes and Multi-Locus Sequence Typing Profiles

**Figure 1:**
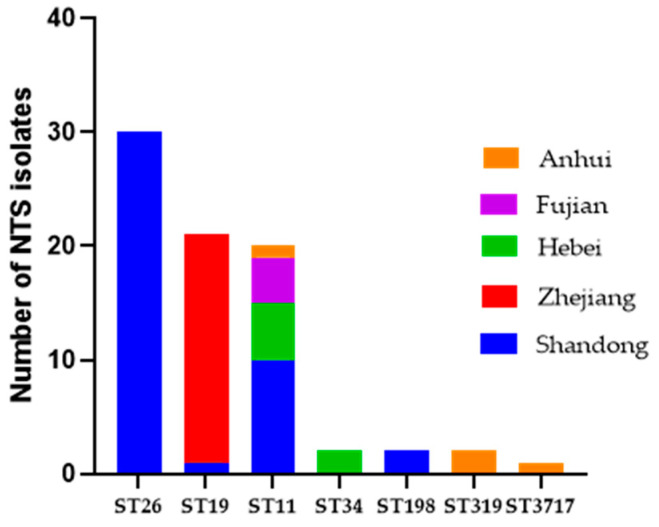
The distribution of different STs among five provinces in China (*n* = 78).

Based on WGS, 78 selected NTS strains represented 6 serovars, with the most frequent serovars being Thompson (*n* = 30) and Typhimurium (*n* = 23) followed by Enteritidis (*n* = 20), Kentucky (*n* = 2), and Tennessee (*n* = 2) (Supplementary Material Table S1). Multi-locus sequence typing (MLST) analysis revealed seven STs, with ST26 (30/78, 38.46%), ST19 (21/78, 26.92%), and ST11 (20/78, 25.64%) being the most prominent (Figure 1). Moreover, ST11 isolates were recovered from Anhui, Fujian, Hebei, and Shandong Provinces, while four serotypes (*S*. Thompson, *S*. Typhimurium, *S*. Enteritidis, and *S*. Kentucky) were isolated from Shandong Province. Furthermore, the *Salmonella* isolates collected in 2021 had more complex serovars (i.e., Enteritidis, Typhimurium, Kentucky, Tennessee, and Gallinarum) than those collected in 2020 (i.e., Enteritidis, Thompson, and Typhimurium). Each serovar represented only one ST, except the *S*. Typhimurium strains, which were divided into ST19 (*n* = 21) and ST34 (*n* = 2) (Supplementary Materials Table S2).

### 2.2. Phenotypic Antimicrobial Resistance

**Figure 2:**
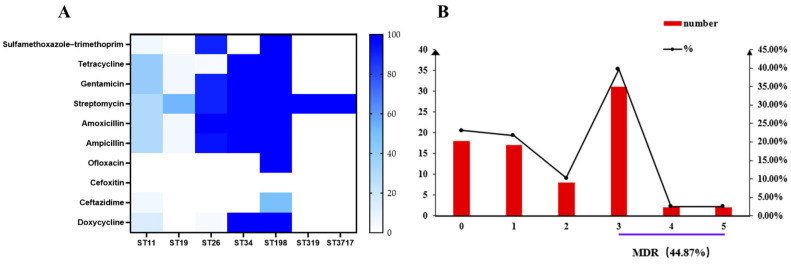
Phenotypic antimicrobial susceptibility of studied isolates. (**A**) The resistance of tested isolates grouped by ST to the tested ten antimicrobial agents from five classes. (**B**) Distribution of MDR among the studied isolates.

The results regarding the AMR of the different ST strains revealed different levels of resistance to 10 antimicrobial agents from five classes (Figure 2A). Overall, 76.92% (60/78) of the isolates were resistant to at least one of the ten antimicrobial drugs tested. The most prevalent resistant phenotypes were streptomycin (65.38%, 51/78), amoxicillin (52.56%, 41/78), ampicillin (50%, 39/78), and sulfamethoxazole–trimethoprim (43.59%, 34/78). However, low levels of resistance were also observed to doxycycline (10.26%, 8/78), ofloxacin (2.56%, 2/78), and ceftazidime (2.56%, 2/78). All the tested strains of the seven STs were susceptible to cefoxitin. Additionally, the ST11 and ST198 isolates showed different levels of resistance to four out of the five antimicrobial classes, while the ST19, ST319, and ST3717 strains were resistant only to the aminoglycoside antimicrobial agents. Notably, the ST198 isolates were resistant to nine antimicrobials, and two *S*. Kentucky strains showed resistance to ofloxacin. Lastly, 44.87% (35/78) of the isolates showed MDR phenotypes that were resistant to at least three classes of antimicrobials (Figure 2B).

### 2.3. Genotypic Antimicrobial Resistance Profiles

Overall, 37 ARGs were identified based on the WGS analysis (Supplementary Material Table S1). The analysis indicated that all 78 NTS strains contained ARGs that are resistant against the following 11 antibiotic classes: aminoglycosides, including *aac(3)-Id* (2.56%, 2/78), *aac(3)-IId* (41.03%, 32/78), *aac(3)-IV* (2.56%, 2/78), *aadA2* (38.46%, 30/78), *aadA7* (2.56%, 2/78), *aadA17* (2.56%, 2/78), *aadA22* (2.56%, 2/78), *aph(3′)-Ia* (5.13%, 4/78), *aph(3″)-Ib* (2.56%, 2/78), *aph(4)-Ia* (2.56%, 2/78), *aph(6)-Id* (2.56%, 2/78), *armA* (38.46%, 30/78), and *rmtB* (2.56%, 2/78); rifampicin, including *ARR-2* (2.56%, 2/78) and *ARR-3* (2.56%, 2/78); beta-lactamases, including *bla*CTX-M-55 (2.56%, 2/78), *bla*CTX-M-65 (2.56%, 2/78), *bla*OXA-10 (2.56%, 2/78), *bla*TEM-1B (46.15%, 36/78), *bla*TEM-70 (2.56%, 2/78), and *bla*TEM-214 (2.56%, 2/78); phenicols, including *cmlA1* (2.56%, 2/78) and *floR* (5.13%, 4/78); trimethoprims, including *dfrA12* (38.46%, 30/78), *dfrA14* (5.13%, 4/78), and *dfrG* (1.28%, 1/78); macrolides, including *erm(B)* (1.28%, 1/78), *mph(A)* (2.56%, 2/78), and *lsa(A)* (1.28%, 1/78); fosfomycins, including *fosA3* (2.56%, 2/78); lincomycins, including *lnu(F)* (5.13%, 4/78); quinolones, including *qnrS1* (5.13%, 4/78); sulfonamides, including *sul1* (41.03%, 32/78) and sul2 (7.69%, 6/78); and tetracyclines, including *tet*(A) (8.97%, 7/78), *tet(B)* (2.56%, 2/78), and *tet(L)* (1.28%, 1/78) (Supplementary Material Figure S1). Meanwhile, the most frequent AMR gene profile was *aac(3)-IId*-*aadA2*-*armA*-*bla*_TEM-1B_-*dfrA12*-*sul1*, which was identified in 30 of the *Salmonella* isolates.

Only two ST198 isolates (2.56%, 2/78) showed phenotypic resistance to ofloxacin, while 42 (53.85%) of the strains presented chromosomal structural gene mutations in the *gyrA* gene (Supplementary Material Table S1). The *parC* point mutation was detected in the quinolone-resistance-determining region of all 30 of the ST26 isolates and both of the ST319 isolates (Figure S2).

The WGS analysis also revealed five different plasmid types in the 78 *Salmonella* isolates (Supplementary Material Table S1), namely IncHI2A, IncHI2, IncFII(S), IncFIB(S), and ColpVC. The most prevalent plasmid replicons among all the plasmids were IncFIB(S) (52.46%, 42/78) and IncFII(S) (52.46%, 42/78). The plasmids IncFIB(S) and IncFII(S) were found to be harbored by 100% of the *S*. Typhimurium isolates and 91% of the *S*. Enteritidis isolates (Figure S3).

### 2.4. Prediction of Virulence Genes

In total, 135 virulence genes representing different virulence pathogenicity mechanisms were identified in the WGS analysis, and the carried rate of 104 virulence genes among the 78 NTS strains was 100% (Figure S4). All 78 strains carried *invABCEFGHIJ* genes, which are used for encoding the invasion of host cells. Notably, our results showed that, except for the ST26 strains, all of the tested strains harbored the virulence plasmid genes (*spvB*, *spvC*, and *spvR*) involved in intra-macrophage survival.

### 2.5. Whole-Genome SNP Analysis of Salmonella Strains

**Figure 3:**
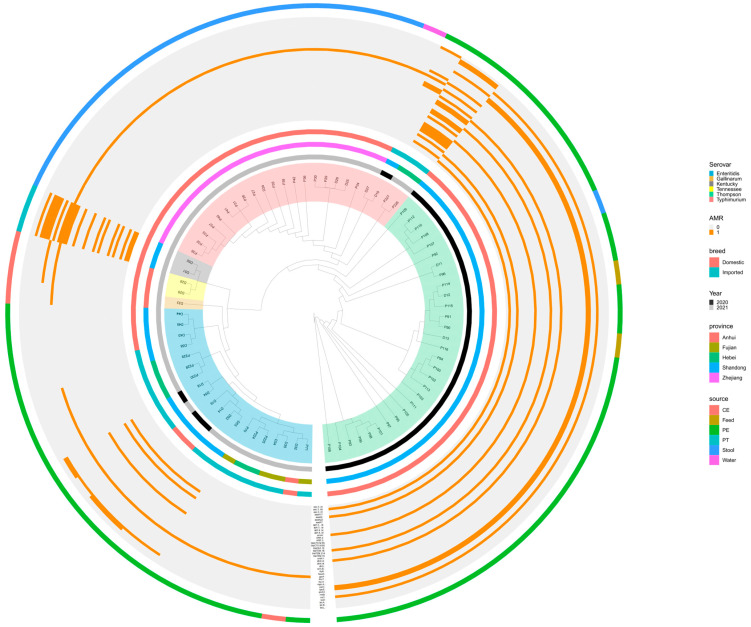
The phylogenomic relationships among 78 NTS strains. Different sources, serovars, years of isolation, regions, breeds, and antimicrobial resistance genes are indicated with different colors.

Whole-genome SNP analysis was employed to probe the deep phylogenetic relationship between the 78 NTS strains (Figure 3). The analysis showed that the isolates belonging to the same serotypes are very closely related to each other. Moreover, the analysis revealed that the closely related *S*. Enteritidis strains were from different provinces.

## 3. Discussion

Non-typhoidal salmonellosis is a dangerous foodborne disease that causes enormous economic loss and threatens public health worldwide [35,36]. The consumption of food, especially poultry or poultry products, contaminated with NTS is the chief cause of salmonellosis in humans. WGS has been confirmed as a functional, cost-effective approach for providing in-depth data on the AMR and virulence genes of *Salmonella* isolates recovered from the poultry industry [37,38]. In our study, WGS analysis was used to evaluate the molecular characteristics of NTS isolates recovered from breeder chicken farms in several Chinese provinces from June 2020 to July 2021. Our results can be combined with information obtained from the monitoring of foodborne diseases in relation to public health.

In our study, 78 NTS strains were grouped into six serovars with seven STs (ST26, ST11, ST19, ST34, ST198, ST319, and ST3717). The dominant serovars were *S*. Thompson-ST26. In contrast, Elbediwi et al. reported that *S*. Enteritidis was the major serotype obtained from dead chick embryos in China’s Henan Province [18]. Compared with the distribution of NTS among the different chicken breeders, we found that *Salmonella* serotypes were more diverse in domestic breeds than in imported ones. The distribution of serovars among imported breeds was primarily consistent with Typhimurium and Enteritidis, while all six serovars with seven STs were detected in the domestic breeds. At the same time, the distribution of NTS serovars varied between 2020 and 2021. Our findings also suggest that the risk of the transmission of zoonotic pathogens may increase owing to rapid economic development in China and that increasingly more *Salmonella* serovars may be detected in the country [39]. In our study, we examined the antimicrobial patterns of 78 NTS from six serotypes—*S*. Enteritidis, Thompson, Typhimurium, Kentucky, Tennessee, and Gallinarum—five of which are generally associated with human and animal infections [40,41,42,43]. Among these, *S*. Gallinarum was previously found to be a predominant serotype in chickens in China and South Korea [44,45,46].

Among the 23 WGS *S*. Typhimurium isolates, 91.30% of the strains were predicted as ST19 and 8.70% were ST34, which demonstrates how WGS technology can help to better distinguish STs. *S*. Typhimurium and Enteritidis are considered to be the two most common serovars responsible for NTS infections in the world. Chen et al. found that *S*. Typhimurium was a prevalent serotype and grew rapidly among children younger than 14 years old in Chian’s Fujian Province between 2012 and 2021 [47], and similar findings were found in another study in China [36]. In addition to that, *S*. Typhimurium and Enteritidis have been isolated from chickens in Malaysia and Turkey [48,49]. Indeed, *S*. Typhimurium has been isolated from poultry and livestock worldwide, which could be the principal hosts for its transmission to humans. Meanwhile, cross-contamination with *S*. Enteritidis may occur through the contamination of meat, water, and eggs [50]. Typically, the Enteritidis serovar colonizes tissues in the reproductive system and increases the threat of fetal infection [50]. Thus, implementing control measures, such as the continuous monitoring of NTS serotypes as well as further controlling these microorganisms within the poultry industry, is necessary.

Previous studies have recognized AMR *Salmonella* isolates as a crucial public health threat [51,52]. Overall, most of the isolates in our study (76.92%) were resistant to at least one of the 10 antimicrobial drugs tested, which is a higher rate than that reported in Vietnam [53]. Furthermore, the most prevalent resistant phenotypes were streptomycin and amoxicillin, which are common antimicrobials used against bacterial infection in animals worldwide [54,55]. These findings are consistent with the findings of Diaz et al. and Tan et al. [56,57]. It seems that the continued use of antimicrobials in the livestock and poultry industries may have resulted in persistent selection pressure on *Salmonella* and, in turn, increased the risk of antimicrobial-resistant strains by enhancing their survival.

High rates of resistance to streptomycin (14–62%) have also been reported in Malaysia, Thailand, and Vietnam [58]. In contrast, *Salmonella* strains recovered from buffalos and pigs tested in Laos showed lower levels of resistance to 10 antibiotic agents, possibly because these animals are primarily raised on private farms [59]. For this reason, *S*. isolates continue to remain sensitive to antimicrobial agents.

Quinolones and third-generation cephalosporins are considered to be important antibiotics for treating *Salmonella* infections, and resistance to the third-generation cephalosporins of strains isolated from retail meat has been found in several developed and developing countries [60,61,62,63]. However, all 78 *Salmonella* strains in our study were sensitive to cefoxitin. One possible reason for this is that antimicrobials have been banned in China as feed additives for food-producing animals since 2020. Several studies have shown that few NTS strains are resistant to quinolone antibiotics [64,65,66]. Of particular concern, *S*. Kentucky (ST198) isolates were found to be resistant to ofloxacin in our study. In addition, the high AMR rate and widespread MDR profiles of *S*. Kentucky have presented risks for public health in recent years [67,68,69]. Our findings suggest that the risk posed by *S*. Kentucky to public health may be especially high. Therefore, the continuous monitoring of, and additional studies on, the AMR of NTS are necessary, and studies are also needed to explore the relationships between *S*. Kentucky from different sources in China.

Because streptomycin has been used to treat human and animal *Salmonella* infections since the 1940s [70,71], the resistance of *Salmonella* spp. to streptomycin has likely increased globally [72,73,74]. Therefore, the detection of *S*. isolates with streptomycin resistance is a critical task for AMR monitoring programs. Our findings indicated that the most prevalent resistant phenotype was streptomycin (65.38%, 51/78), which aligns with the results of several previous studies [75,76]. For instance, Long et al. also reported that 90.8% of isolates exhibited high resistance to streptomycin [77].

AMR has emerged as a major problem for public health in the 21st century and one that threatens the effective treatment of diseases caused by bacteria. Majowicz et al. have reported that each year, there are 94 million cases of NTS gastroenteritis that result in 155,000 deaths globally, and that the majority of the disease burden is in Southeast Asia [78]. A high prevalence (44.87%) of MDR non-typhoidal *Salmonella* isolates was also observed in our study. This finding is consistent with the results of a previous study on various host species in the United States [79] but lower than the prevalence found in MDR *S*. strains isolated from blood samples in Chattogram City, Bangladesh [80]. The difference may relate to the abuse of drugs in the poultry breeding industry. Indeed, the unreasonable or excessive use of antimicrobials in the veterinary industry has resulted in the development of MDR strains that seriously threaten public health.

Because China heavily relies on poultry meat and eggs as crucial sources of dietary protein for human consumption, *Salmonella* and related resistance genes pose a significant threat to both the poultry industry and food safety in the country, which, in turn, jeopardize public health. After comparing gene annotations with the Resfinder database, we observed a drastic decrease in *aadA2*, *aac(3)-IId*, *armA*, *bla*_TEM-1B_, *dfrA12*, and *sul1* from 2020 to 2021 (88–97% vs. 0–7%). Furthermore, multiple ARGs were found in strains from Shandong and Hebei Provinces, whereas no ARGs were detected in the strains from Zhejiang, Anhui, or Fujian Provinces. Meanwhile, a very large difference was found between the strains from different host breeds and sample types; strains from feed and parental embryos carried far more ARGs than those from stool and commercial embryos, which indicates that feed may be an important source of ARG contamination. Our findings also revealed the high prevalence of aminoglycoside and β-lactame genes in *S*. Thompson strains, including *aadA2*, *aac(3)-IId*, and *bla*TEM-1B, which is consistent with other recent findings [81].

The number of human infections with the MDR *S*. Kentucky has increased significantly in many developed countries in recent years [82]. In Europe in 2016, the most common serovars of 1721 isolates in broilers and 663 isolates in turkeys showed more overlap than in Canada, with Kentucky serovars among the major serovars in both broilers and turkeys [83]. The high number of resistance genes found in two *S*. Kentucky isolates was not unexpected in light of other reports [84,85]. Based on the above, Kentucky serovars should be regularly monitored and controlled to reduce their risk to public health. Comprehensive prevention and control measures should also be established on poultry farms.

Although phenotypic and genotypic resistance are highly consistent, inconsistencies are possible. In our study, thirty-six NTS isolates harbored β-lactame resistance genes but were phenotypically susceptible. However, the presence of resistance genes in genomes does not necessarily lead to phenotypic resistance [86]. Indeed, antimicrobial resistance is determined not only by the presence or absence of resistance genes. Other mechanisms, including enzyme activation, target modification or protection, the regulation of antimicrobial-resistant gene expression, and even changes in the cell wall, can play important roles in antimicrobial resistance [86,87]. Thus, using both phenotypic and genotypic methods is essential for detecting the studied isolates.

CTX-M genes have been disseminated worldwide, and among their many variants, CTX-M-15 and CTX-M-14 are the most prevalent [88,89]. CTX-M-55, a CTX-M-15 variant, is considered to be a major extended-spectrum β-lactamase (ESBL) gene. In our study, we detected two ST198 (*S*. Kentucky) isolates collected from *Salmonella*-infected breeders that carried the *bla*CTX-M-55 ESBL gene. Moreover, these two strains were not entirely sensitive to ofloxacin nor did they harbor quinolone-resistance genes. Our findings thus confirm that the coexistence or co-transfer of PMQR genes in CTX-M-producing *Salmonella* strains may increase their probability of survival in the presence of quinolones [90].

In our study, plasmid replicons were detected in 100% of the NTS strains, and 96.15% of the strains carried at least two replicon types. Mobilizable plasmids (i.e., IncHI2) were detected in the multidrug-resistant strains, which play an important role in the transmission of resistance in *Salmonella* spp. As past research has shown, the activity of multiple mobile genetic elements may contribute to antibiotic resistance evolution and dissemination between different plasmid replicons [91]. Additionally, IncFIB(S) and IncFII(S), which are both encoding virulence factors, were identified in *S*. Enteritidis (18 isolates) and *S*. Typhimurium (21 isolates). These two plasmids have been previously detected in *S*. Enteritidis isolates from farms, slaughterhouses, and markets in China’s Xinjiang Province [86] as well as in *S*. Typhimurium isolated from an animal hospital in Hangzhou, China [92].

Bacterial pathogenicity is closely linked to its virulence genes [93]. The *spv* genes, located on the virulence plasmids of *S*. *enterica*, cause non-typhoidal bacteremia [94]. In this study, other than ST26, all strains tested harbored the virulence plasmid genes (*spvB*, *spvC*, and *spvR*) involved in intra-macrophage survival, possibly due to differences in the pathogenic potential of different *Salmonella* serovars. Our results show that adhesion-related genes (*ace*) only exist in *S.* Thompson, whereas the detection rates of T3SS-associated virulence genes (*inv*, *spa*, *org*, *ssa*, *ssc*, and *sse*) varied among the serovars.

Finally, when analyzing the phylogenetic tree for the 78 *Salmonella* genomes based on SNP alignment, the tree’s branch structures were consistent with the serovars and STs. The co-occurrence of ARGs and serotypes indicated a possible host preference among the ARGs.

## 4. Materials and Methods

### 4.1. Salmonella Strains

Our study was conducted using 78 NTS strains selected from 440 *Salmonella* strains that were isolated from 7534 samples from 40 breeder chicken farms in 11 Chinese provinces from June 2020 to July 2021. Of those strains, 34 isolates recovered from 2020 and 44 from 2021 were used in the study. The tested isolates were selected from different breeds, farms, and provinces (Supplementary Material Table S1). The isolation, identification, and serotyping of the *Salmonella* strains were performed according to our previously described methods [95]. Briefly, each sample was added to 4.5 mL of Buffered Peptone Water (Qingdao Hope Bio-technology Co., Ltd., Qingdao, China) and cultured at 37 °C for 8–12 h. Next, 0.5 mL of enriched culture was inoculated into 4.5 mL of Selenite Cystine Broth (Qingdao Hope Bio-Technology Co., Ltd., Qingdao, China) and 4.5 mL of Tetrathionate Broth Base (Qingdao Hope Bio-Technology Co., Ltd., Qingdao, China). Next, the cultures were inoculated on a Xylose Lysine Desoxycholate Agar base (Qingdao Hope Bio-technology Co., Ltd., Qingdao, China) and hatched at 37 °C for 24–48 h. Colonies were confirmed by polymerase chain reaction assays. All strains were isolated and identified by the Veterinary Public Health Laboratory of Shandong Agricultural University.

### 4.2. Antimicrobial Resistance Tests

The antimicrobial susceptibility of the studied strains was tested according to the Kirby–Bauer disk diffusion method and the recommendations of the Clinical Laboratory Standards Institute [96]. The test involved 10 antimicrobial agents belonging to the following five classes: β-lactames, which included ampicillin (10 µg), amoxicillin (20 µg), ceftazidime (µg), and cefoxitin (30 µg); tetracyclines, which included tetracycline (30 µg) and doxycycline (DOX, 30 µg); quinolones, including ofloxacin (5 µg); aminoglycosides, which included gentamicin (10 µg) and streptomycin (10 µg); and sulfonamides, including sulfamethoxazole–trimethoprim (25 µg). MDR bacteria were defined as having acquired non-susceptibility to at least one agent in three or more antimicrobial categories. *Escherichia coli* ATCC 25922 was used as a quality control strain.

### 4.3. Whole-Genome Sequencing

The genomic DNA from all 78 isolates was extracted using a Genomic DNA Purification Kit (Promega Biotech Co., Ltd., Beijing, China) and sequenced on the Illumina NovaSeq 6000 platform (Illumina Scientific Co., Ltd., Shanghai, China). These readings underwent a quality control procedure via FastQC and were assembled with SPAdes v.3.15.4 [97]. The resulting assemblies were then deposited at the National Center for Biotechnology Information under the BioProject accession number PRJNA976535.

### 4.4. Gene Annotation and Analysis

The assemblies were serotyped using sistr-cmd v.1.1.1 [98] and screened against the *Salmonella* seven-locus MLST database, Resfinder database, PlasmidFinder database, and Pointfinder database using Staramr v.2.0.1 [99] in order to assign STs and detect acquired antimicrobial resistance genes, plasmid replicons, and point mutations.

The results were transformed into a binary table in R v.3.6.0 to analyze the presence or absence of acquired ARG alleles, and the prevalence of each gene in the different isolates was grouped by serotypes or background information. All assemblies were annotated using Prokka v.1.14.6 [100] and subjected to a pangenome analysis using Roary v.3.12.0 [101].

## 5. Conclusions

To our knowledge, our study is the first to use a WGS approach to investigate the molecular epidemiological characteristics of NTS strains isolated from breeder chicken farms in five provinces in China between 2020 and 2021. Our study shows that most NTS isolates harbor different antimicrobial resistance genes, as determined by plasmids and chromosomes, in addition to virulence genes associated with multiple pathogenic mechanisms, both of which carry zoonotic risk.

## Data Availability

The datasets presented in this study can be found in online repositories. The names of the repository/repositories and accession number(s) can be found via the NCBI under the BioProject accession number PRJNA976535.

## Supplementary Materials

The following supporting information can be downloaded at: https://www.mdpi.com/article/10.3390/antibiotics12111642/s1, Table S1: The Information for 78 NTS isolates strains in this study. Table S2: Distribution of serotypes and MLST patterns for *Salmonella* isolates (*n* = 78). Figure S1: The heatmap of antimicrobial resistance genes in the studied NTS isolates. Figure S2: The heatmap of chromosomic mutations in NTS isolates. Figure S3: The heatmap of plasmid distribution in *Salmonella* isolates. Figure S4: Virulence gene detection based on WGS of *Salmonella* strains.
