# Supplementary material for: Whole-Genome Sequencing Analysis of Non-Typhoidal Salmonella Isolated from Breeder Poultry Farm Sources in China, 2020–2021
Source: Antibiotics (Basel). 2023 Nov 19;12(11):1642. doi: 10.3390/antibiotics12111642 (PMC10669045; doi:10.3390/antibiotics12111642)
Supplement: Supplementary file 1 [file antibiotics-12-01642-s001.zip › antibiotics-2698784-supplementary.pdf]

Table S1. The Information for 78 NTS isolates strains in this study.

Numbers	Years	Regions	Sources	Breeds	Domestic or Imported Breeds	Serotypes	Antimicrobial Resistance Patterns*	Antimicrobial Resistance Genes	Plasmids	MLST
20SD01	2020	Shandong	Feed	Langya	Domestic breeds	*S*. Thompson	SXT-AMX-STR-GM-AMP	*aac(3)-Iid, aadA2, armA, bla*TEM-1B, *dfrA12, parC, qacE, sul1*	IncHI2, IncHI2A	26
20SD02	2020	Shandong	Feed	Langya	Domestic breeds	*S*. Thompson	SXT-AMX-STR-GM-AMP	*aac(3)-Iid, aadA2, armA, bla*TEM-1B, *dfrA12, parC, qacE, sul1*	ColpVC, IncHI2, IncHI2A	26
20SD03	2020	Shandong	Stool	Langya	Domestic breeds	*S*. Thompson	SXT-AMX-STR-GM-AMP	*aac(3)-IId, aadA2, armA, bla*TEM-1B, *dfrA12, parC, qacE, sul1*	IncHI2, IncHI2A	26
20SD04	2020	Shandong	Parental embryos	Langya	Domestic breeds	*S*. Thompson	SXT-AMX-STR-GM-AMP	*aac(3)-IId, aadA2, armA, bla*TEM-1B, *dfrA12, parC, qacE, sul1*	IncHI2, IncHI2A	26
20SD05	2020	Shandong	Parental embryos	Langya	Domestic breeds	*S*. Thompson	SXT-AMX-STR-GM-AMP	*aac(3)-IId, aadA2, armA, bla*TEM-1B, *dfrA12, parC, qacE, sul1*	IncHI2, IncHI2A	26
20SD07	2020	Shandong	Parental embryos	Langya	Domestic breeds	*S*. Thompson	SXT-AMX-STR-GM-AMP	*aac(3)-IId, aadA2, armA, bla*TEM-1B, *dfrA12, parC, qacE, sul1*	IncHI2, IncHI2A	26
20SD09	2020	Shandong	Parental embryos	Langya	Domestic breeds	*S*. Thompson	SXT-AMX-STR-GM-AMP	*aac(3)-IId, aadA2, armA, bla*TEM-1B, *dfrA12, parC, qacE, sul1*	IncHI2, IncHI2A	26
20SD10	2020	Shandong	Parental embryos	Langya	Domestic breeds	*S*. Thompson	SXT-AMX-TET-DOX-STR-GM-AMP	*aac(3)-IId, aadA2, armA, bla*TEM-1B, *dfrA12, parC, qacE, sul1*	IncHI2, IncHI2A	26
20SD11	2020	Shandong	Parental embryos	Langya	Domestic breeds	*S*. Thompson	SXT-AMX-STR-GM	*aac(3)-IId, aadA2, armA, bla*TEM-1B, *dfrA12, parC, qacE, sul1*	IncHI2, IncHI2A	26
20SD12	2020	Shandong	Parental embryos	Langya	Domestic breeds	*S*. Thompson	SXT-AMX-STR-GM-AMP	*aac(3)-IId, aadA2, armA, bla*TEM-1B, *dfrA12, parC, qacE, sul1*	IncHI2, IncHI2A	26
20SD13	2020	Shandong	Parental embryos	Langya	Domestic breeds	*S*. Thompson	SXT-AMX-STR-GM-AMP	*aac(3)-IId, aadA2, armA, bla*TEM-1B, *dfrA12, parC, qacE, sul1*	IncHI2, IncHI2A	26

20SD14	2020	Shandong	Parental embryos	Langya	Domestic breeds	*S*. Thompson	SXT-AMX-STR-GM	*aac(3)-IId*, *aadA2*, *armA*, *bla*TEM-1B, *dfrA12*, *parC*, *qacE*, *sul1*	IncHI2, IncHI2A	26
20SD15	2020	Shandong	Parental embryos	Langya	Domestic breeds	*S*. Thompson	SXT-AMX-STR-GM-AMP	*aac(3)-IId*, *aadA2*, *armA*, *bla*TEM-1B, *dfrA12*, *parC*, *qacE*, *sul1*	IncHI2, IncHI2A	26
20SD16	2020	Shandong	Parental embryos	Langya	Domestic breeds	*S*. Thompson	SXT-AMX-STR-GM-AMP	*aac(3)-IId*, *aadA2*, *armA*, *bla*TEM-1B, *dfrA12*, *parC*, *qacE*, *sul1*	IncHI2, IncHI2A	26
20SD17	2020	Shandong	Parental embryos	Langya	Domestic breeds	*S*. Thompson	SXT-AMX-GM-AMP	*aac(3)-IId*, *aadA2*, *armA*, *bla*TEM-1B, *dfrA12*, *parC*, *qacE*, *sul1*	IncHI2, IncHI2A	26
20SD18	2020	Shandong	Parental embryos	Langya	Domestic breeds	*S*. Thompson	AMX-STR-GM-AMP	*aac(3)-IId*, *aadA2*, *armA*, *bla*TEM-1B, *dfrA12*, *parC*, *qacE*, *sul1*	IncHI2, IncHI2A	26
20SD19	2020	Shandong	Parental embryos	Langya	Domestic breeds	*S*. Thompson	SXT-AMX-STR-GM-AMP	*aac(3)-IId*, *aadA2*, *armA*, *bla*TEM-1B, *dfrA12*, *parC*, *qacE*, *sul1*	IncHI2, IncHI2A	26
20SD20	2020	Shandong	Parental embryos	Langya	Domestic breeds	*S*. Thompson	SXT-AMX-STR-GM-AMP	*aac(3)-IId*, *aadA2*, *armA*, *bla*TEM-1B, *dfrA12*, *parC*, *qacE*, *sul1*	IncHI2, IncHI2A	26
20SD21	2020	Shandong	Parental embryos	Langya	Domestic breeds	*S*. Thompson	SXT-AMX-STR-GM-AMP	*aac(3)-IId*, *aadA2*, *armA*, *bla*TEM-1B, *dfrA12*, *parC*, *qacE*, *sul1*	IncHI2, IncHI2A	26
20SD22	2020	Shandong	Parental embryos	Langya	Domestic breeds	*S*. Thompson	SXT-AMX-STR-GM-AMP	*aac(3)-IId*, *aadA2*, *armA*, *bla*TEM-1B, *dfrA12*, *parC*, *qacE*, *sul1*	IncHI2, IncHI2A	26
20SD23	2020	Shandong	Parental embryos	Langya	Domestic breeds	*S*. Thompson	SXT-AMX-STR-GM-AMP	*aac(3)-IId*, *aadA2*, *armA*, *bla*TEM-1B, *dfrA12*, *parC*, *qacE*, *sul1*	IncHI2, IncHI2A	26
20SD24	2020	Shandong	Parental embryos	Langya	Domestic breeds	*S*. Thompson	SXT-AMX-STR-GM-AMP	*aac(3)-IId*, *aadA2*, *armA*, *bla*TEM-1B, *dfrA12*, *parC*, *qacE*, *sul1*	IncHI2, IncHI2A	26
20SD25	2020	Shandong	Parental embryos	Langya	Domestic breeds	*S*. Thompson	SXT-AMX-STR-GM-AMP	*aac(3)-IId*, *aadA2*, *armA*, *bla*TEM-1B, *dfrA12*, *parC*, *qacE*, *sul1*	ColpVC, IncHI2, IncHI2A	26

20SD26	2020	Shandong	Parental embryos	Langya	Domestic breeds	*S*. Thompson	SXT-AMX-STR-GM-AMP	*aac(3)-IId*, *aadA2*, *armA*, *bla*TEM-1B, *dfrA12*, *parC*, *qacE*, *sul1*	IncHI2, IncHI2A	26
20SD27	2020	Shandong	Parental embryos	Langya	Domestic breeds	*S*. Thompson	SXT-AMX-STR-GM-AMP	*aac(3)-IId*, *aadA2*, *armA*, *bla*TEM-1B, *dfrA12*, *parC*, *qacE*, *sul1*	IncHI2, IncHI2A	26
20SD28	2020	Shandong	Parental embryos	Langya	Domestic breeds	*S*. Thompson	SXT-AMX-STR-GM-AMP	*aac(3)-IId*, *aadA2*, *armA*, *bla*TEM-1B, *dfrA12*, *parC*, *qacE*, *sul1*	IncHI2, IncHI2A	26
20SD29	2020	Shandong	Parental embryos	Langya	Domestic breeds	*S*. Thompson	SXT-AMX-GM-AMP	*aac(3)-IId*, *aadA2*, *armA*, *bla*TEM-1B, *dfrA12*, *parC*, *qacE*, *sul1*	IncHI2, IncHI2A	26
20SD30	2020	Shandong	Parental embryos	Langya	Domestic breeds	*S*. Thompson	SXT-AMX-AMP	*aac(3)-IId*, *aadA2*, *armA*, *bla*TEM-1B, *dfrA12*, *parC*, *qacE*, *sul1*	IncHI2, IncHI2A	26
20SD31	2020	Shandong	Parental embryos	Langya	Domestic breeds	*S*. Thompson	AMX-STR-AMP	*aac(3)-IId*, *aadA2*, *armA*, *bla*TEM-1B, *dfrA12*, *parC*, *qacE*, *sul1*	IncHI2, IncHI2A	26
20SD32	2020	Shandong	Parental embryos	Langya	Domestic breeds	*S*. Thompson	AMX-STR-AMP	*aac(3)-IId*, *aadA2*, *armA*, *bla*TEM-1B, *dfrA12*, *parC*, *qacE*, *sul1*	IncHI2, IncHI2A	26
20JN04	2020	Shandong	Parental embryos	Bairi	Domestic breeds	***S***. Enteritidis	TET-GM-AMP-AMX-DOX	*aph(3'')-Ib*, *aph(6)-Id*, *bla*TEM-1B, *gyrA*, *sul2*, *tet(A)*	IncFIB(S), IncFII(S)	11
20JN14	2020	Shandong	Parental embryos	Bairi	Domestic breeds	***S***. Enteritidis	TET-GM-AMP-AMX-DOX	*aph(3'')-Ib*, *aph(6)-Id*, *bla*TEM-1B, *gyrA*, *sul2*, *tet(A)*	IncFIB(S), IncFII(S)	11
20TA01	2020	Shandong	Parental embryos	Hubbard chicken	Imported breeds	***S***. Enteritidis	TET-STR-GM-AMP-AMX-DOX	*aph(3'')-Ib*, *aph(6)-Id*, *bla*TEM-1B, *gyrA*, *sul2*, *tet(A)*	IncFIB(S), IncFII(S)	11
20TA15	2020	Shandong	Parental embryos	Hubbard chicken	Imported breeds	***S***. Enteritidis	GM-AMP-AMX	*dfrG*, *erm(B)*, *gyrA*, *lsa(A)*, *tet(L)*	IncFIB(S), IncFII(S)	19
C104	2021	Zhejiang	Stool	Broiler	Domestic breeds	*S*. Typhimurium	TET-STR	*gyrA*	IncFIB(S), IncFII(S)	19
C108	2021	Zhejiang	Stool	Broiler	Domestic breeds	*S*. Typhimurium	STR	*gyrA*	IncFIB(S), IncFII(S)	19

C111	2021	Zhejiang	Stool	Broiler	Domestic breeds	*S*. Typhimurium	STR	*gyrA*	IncFIB(S), IncFII(S)	19
C113	2021	Zhejiang	Stool	Broiler	Domestic breeds	*S*. Typhimurium		*gyrA*	IncFIB(S), IncFII(S)	19
C121	2021	Zhejiang	Stool	Broiler	Domestic breeds	*S*. Typhimurium		*gyrA*	IncFIB(S), IncFII(S)	19
C122	2021	Zhejiang	Stool	Broiler	Domestic breeds	*S*. Typhimurium		*gyrA*	IncFIB(S), IncFII(S)	19
C129	2021	Zhejiang	Stool	Broiler	Domestic breeds	*S*. Typhimurium	STR	*gyrA*	IncFIB(S), IncFII(S)	19
C130	2021	Zhejiang	Stool	Broiler	Domestic breeds	*S*. Typhimurium	STR	*gyrA*	IncFIB(S), IncFII(S)	19
C145	2021	Zhejiang	Stool	Broiler	Domestic breeds	*S*. Typhimurium	STR	*gyrA*	IncFIB(S), IncFII(S)	19
C151	2021	Zhejiang	Stool	Broiler	Domestic breeds	*S*. Typhimurium	STR	*gyrA*	IncFIB(S), IncFII(S)	19
C162	2021	Zhejiang	Stool	Broiler	Domestic breeds	*S*. Typhimurium	STR	*gyrA*	IncFIB(S), IncFII(S)	19
C170	2021	Zhejiang	Stool	Broiler	Domestic breeds	*S*. Typhimurium		*gyrA*	IncFIB(S), IncFII(S)	19
C182	2021	Zhejiang	Stool	Broiler	Domestic breeds	*S*. Typhimurium		*gyrA*	IncFIB(S), IncFII(S)	19
C185	2021	Zhejiang	Stool	Broiler	Domestic breeds	*S*. Typhimurium		*gyrA*	IncFIB(S), IncFII(S)	19
C190	2021	Zhejiang	Stool	Broiler	Domestic breeds	*S*. Typhimurium	STR	*gyrA*	IncFIB(S), IncFII(S)	19
C191	2021	Zhejiang	Stool	Broiler	Domestic breeds	*S*. Typhimurium	STR	*gyrA*	IncFIB(S), IncFII(S)	19
C192	2021	Zhejiang	Stool	Broiler	Domestic breeds	*S*. Typhimurium	STR	*gyrA*	IncFIB(S), IncFII(S)	19
C195	2021	Zhejiang	Stool	Broiler	Domestic breeds	*S*. Typhimurium		*gyrA*	IncFIB(S), IncFII(S)	19
C197	2021	Zhejiang	Stool	Broiler	Domestic breeds	*S*. Typhimurium		*gyrA*	IncFIB(S), IncFII(S)	19
C205	2021	Zhejiang	Water	Broiler	Domestic breeds	*S*. Typhimurium		*gyrA*	IncFIB(S), IncFII(S)	19

D2	2021	Anhui	Commercial embryos	Partfidge chicken	Domestic breeds	*S*. Tennessee	STR	*gyrA*, *parC*		319
D5	2021	Anhui	Commercial embryos	Partfidge chicken	Domestic breeds	*S*. Tennessee	STR	*gyrA*, *parC*		319
D84	2021	Anhui	Commercial embryos	Partfidge chicken	Domestic breeds	***S***. Enteritidis		*gyrA*	IncFIB(S), IncFII(S)	11
D89	2021	Anhui	Commercial embryos	Partfidge chicken	Domestic breeds	*S*. Gallinarum	STR	*gyrA*	ColpVC	3717
E17	2021	Fujian	Parental embryos	White feather chicken	Imported breeds	*S*. Enteritidis	TET	*gyrA*	IncFIB(S), IncFII(S)	11
E70	2021	Fujian	Parental embryos	White feather chicken	Imported breeds	*S*. Enteritidis		*gyrA*	IncFIB(S), IncFII(S)	11
E86	2021	Fujian	Parental embryos	White feather chicken	Imported breeds	*S*. Enteritidis	TET	*gyrA*	IncFIB(S), IncFII(S)	11
E88	2021	Fujian	Parental embryos	White feather chicken	Imported breeds	*S*. Enteritidis		*gyrA*	IncFIB(S), IncFII(S)	11
K57	2021	Shandong	Parental embryos	Bairi	Domestic breeds	*S*. Enteritidis			IncFIB(S), IncFII(S)	11
K16	2021	Shandong	Parental embryos	Bairi	Domestic breeds	*S*. Enteritidis	STR-CAZ		IncFIB(S), IncFII(S)	11
K6	2021	Shandong	Parental embryos	Bairi	Domestic breeds	*S*. Enteritidis			IncFIB(S), IncFII(S)	11
21JX20	2021	Shandong	Parental tissue	Langya	Domestic breeds	***S***. Kentucky	SXT-TET-GM-STR-AMP-OF-AMX-DOX	*aac(3)-Id*, *aac(3)-IId*, *aadA17*, *aadA7*, *aph(3′)-Ia*, *ARR-2*, *bla*CTX-M-55, *bla*TEM-214, *dfrA14*, *floR*, *fosA3*, *gyrA*, *lnu(F)*, *mph(A)*, *qacE*, *qnrS1*, *rmtB*, *sul1*, *tet(A)*	ColpVC	198
21JX21	2021	Shandong	Parental tissue	Langya	Domestic breeds	***S***. Kentucky	SXT-TET-GM-STR-AMP-OF-AMX-CAZ-DOX	*aac(3)-Id*, *aac(3)-IId*, *aadA17*, *aadA7*, *aph(3′)-Ia*, *ARR-2*, *bla*CTX-M-55, *bla*TEM-214, *dfrA14*, *floR*, *fosA3*, *gyrA*,	ColpVC	198

								lnu(F), *mph(A)*, *qacE*, *qnrS1*, *rmtB*, *sul1*, *tet(A)*		
T14	2021	Shandong	Parental embryos	White feather chicken	Imported breeds	*S*. Enteritidis	SXT-TET-GM-STR-AMP-AMX	*aph(3'')-Ib*, *aph(6)-Id*, *bla*TEM-1B, *gyrA*, *sul2*	IncFIB(S), IncFII(S)	11
T28	2021	Shandong	Parental embryos	White feather chicken	Imported breeds	*S*. Enteritidis	STR-AMP-AMX	*aph(3'')-Ib*, *aph(6)-Id*, *bla*TEM-1B, *gyrA*, *sul2*	IncFIB(S), IncFII(S)	11
T67	2021	Shandong	Parental embryos	White feather chicken	Imported breeds	*S*. Enteritidis	TET-STR-AMP-AMX	*aph(3'')-Ib*, *aph(6)-Id*, *bla*TEM-1B, *gyrA*, *sul2*	IncFIB(S), IncFII(S)	11
21SD18	2021	Shandong	Parental embryos	HY-line brown layer	Imported breeds	*S*. Enteritidis	STR		IncFIB(S), IncFII(S)	11
A1	2021	Hebei	Parental embryos	White feather chicken	Imported breeds	*S*. Enteritidis		*gyrA*	IncFIB(S), IncFII(S)	11
A2	2021	Hebei	Parental embryos	White feather chicken	Imported breeds	*S*. Enteritidis		*gyrA*	IncFIB(S), IncFII(S)	11
B12	2021	Hebei	Parental embryos	White feather chicken	Imported breeds	*S*. Typhimurium	TET-GM-STR-AMP-AMX-DOX	*aadA22*, *aph(3'')-Ib*, *aph(3')-Ia*, *aph(6)-Id*, *ARR-3*, *bla*CTX-M-65, *bla*OXA-10, *bla*TEM-70, *cmlA1*, *dfrA14*, *fosA3*, *lnu(F)*, *qnrS1*, *tet(A)*, *tet(B)*	IncHI2, IncHI2A	34
B28	2021	Hebei	Parental embryos	White feather chicken	Imported breeds	*S*. Typhimurium	TET-GM-STR-AMP-AMX-DOX	*aadA22*, *aph(3'')-Ib*, *aph(3')-Ia*, *aph(6)-Id*, *ARR-3*, *bla*CTX-M-65, *bla*OXA-10, *bla*TEM-70, *cmlA1*, *dfrA14*, *fosA3*, *lnu(F)*, *qnrS1*, *tet(A)*, *tet(B)*	IncHI2, IncHI2A	34
1–5	2021	Hebei	Parental embryos	White feather chicken	Imported breeds	*S*. Enteritidis	TET	*gyrA*	IncFIB(S), IncFII(S)	11
4–15	2021	Hebei	Parental embryos	White feather chicken	Imported breeds	*S*. Enteritidis		*gyrA*	IncFIB(S), IncFII(S)	11

5–15	2021	Hebei	Parental embryos	White feather chicken	Imported breeds	*S*. Enteritidis			*gyrA*	IncFIB(S), IncFII(S)	11

*: ampicillin-AMP, amoxicillin-AMX, ceftazidime- CAZ, cefoxitin-FOX, tetracycline-TET, doxycycline-DOX, ofloxacin-OF, gentamicin-GM, streptomycin -STR, sulfamethoxazole–trimethoprim -SXT.

Table S2. Distribution of serotypes and MLST patterns for *Salmonella* isolates (*n* = 78).

**Serotypes**	**Sample types (no. of isolates)**							**MLST patterns**							
	**Parental dead embryos (*n* =49)**	**Feces (*n* =20)**	**Commercial dead embryos (*n* =4)**	**Food (*n* =2)**	**Parental tissue (*n* =2)**	**Water (*n* =1)**	**Total *n*/*N* (%)**	***aroC***	***dnaN***	***hemD***	***hisD***	***purE***	***sucA***	***thrA***	**STs**
S. Thompson	27	1	0	2	0	0	30/78 (38.46%)	14	13	18	12	14	18	1	26
S. Typhimurium	1	19	0	0	0	1	21/78 (26.92%)	10	7	12	9	5	9	2	19
S. Typhimurium	2	0	0	0	0	0	2/78 (2.56%)	10	19	12	9	5	9	2	34
S. Enteritidis	19	0	1	0	0	0	20/78 (25.64%)	5	2	3	7	6	6	11	11
S. Kentucky	0	0	0	0	2	0	2/78 (2.56%)	76	14	3	77	64	64	67	198
S. Tennessee	0	0	2	0	0	0	2/78 (2.56%)	118	107	8	51	2	117	16	319
S. Gallinarum	0	0	1	0	0	0	1/78 (1.28%)	5	2	361	7	31	668	11	3717

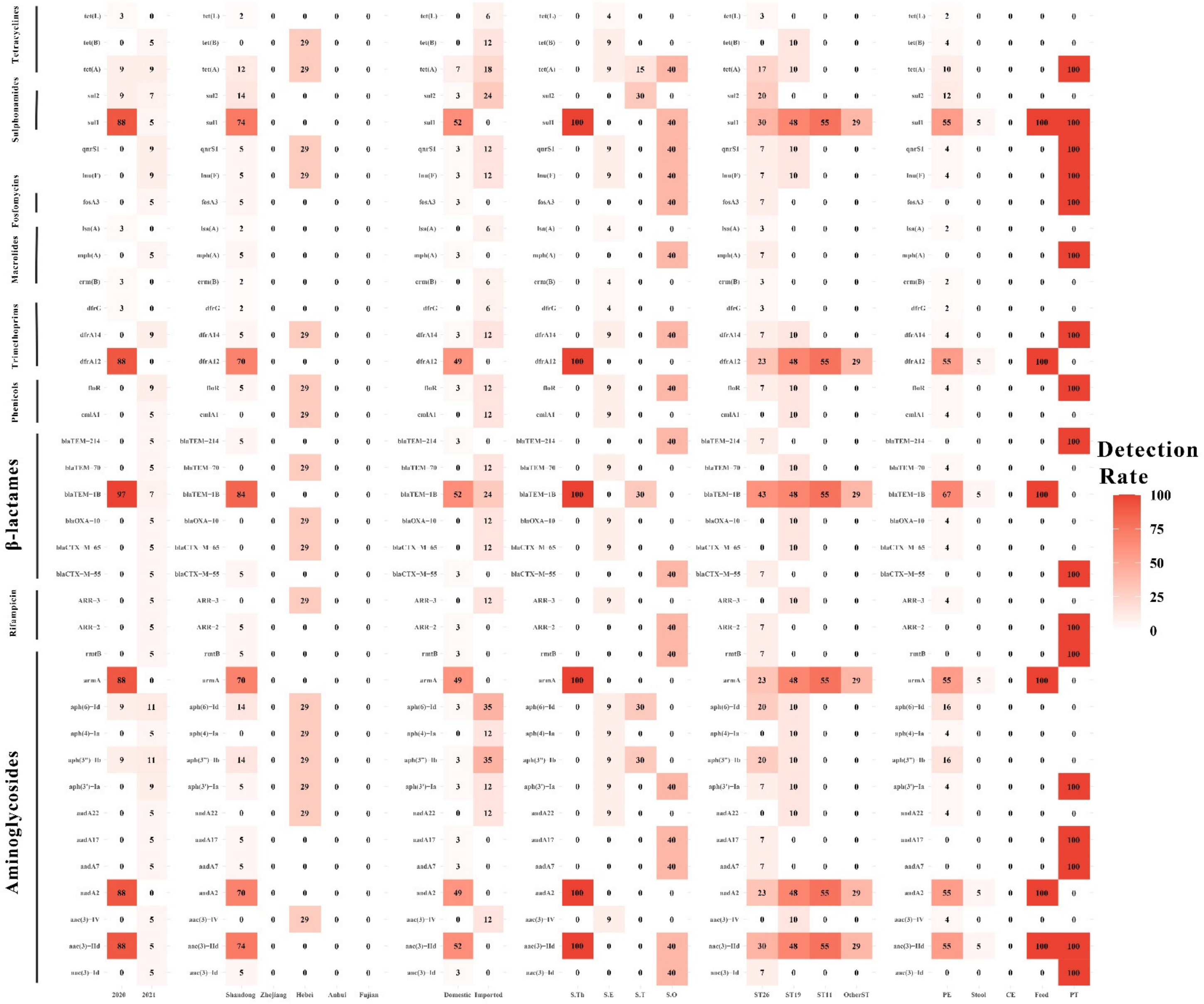

Figure S1. The heatmap of antimicrobial resistance genes in the studied NTS isolates.

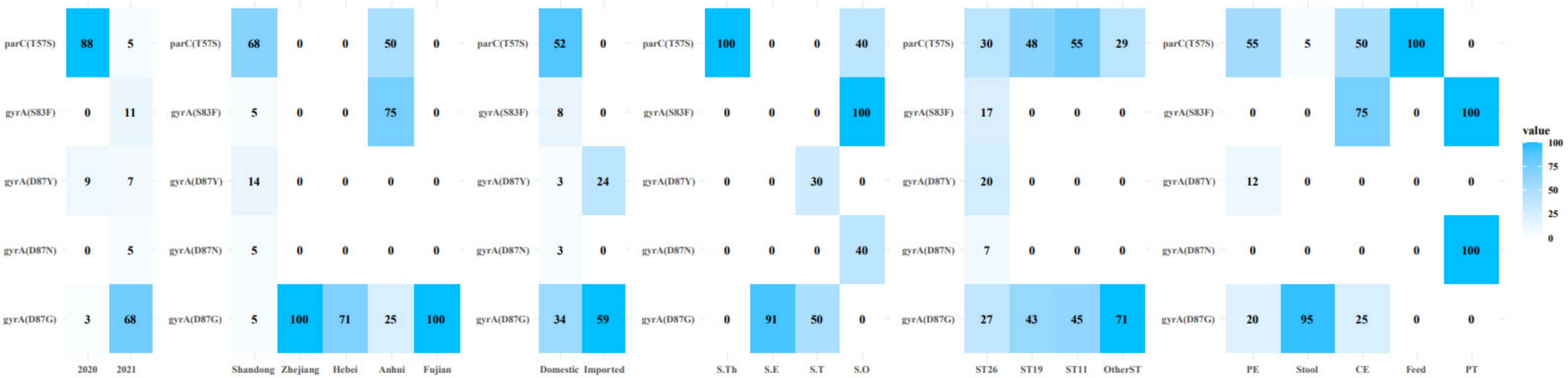

Figure S2. The heatmap of chromosomic mutations in NTS isolates.

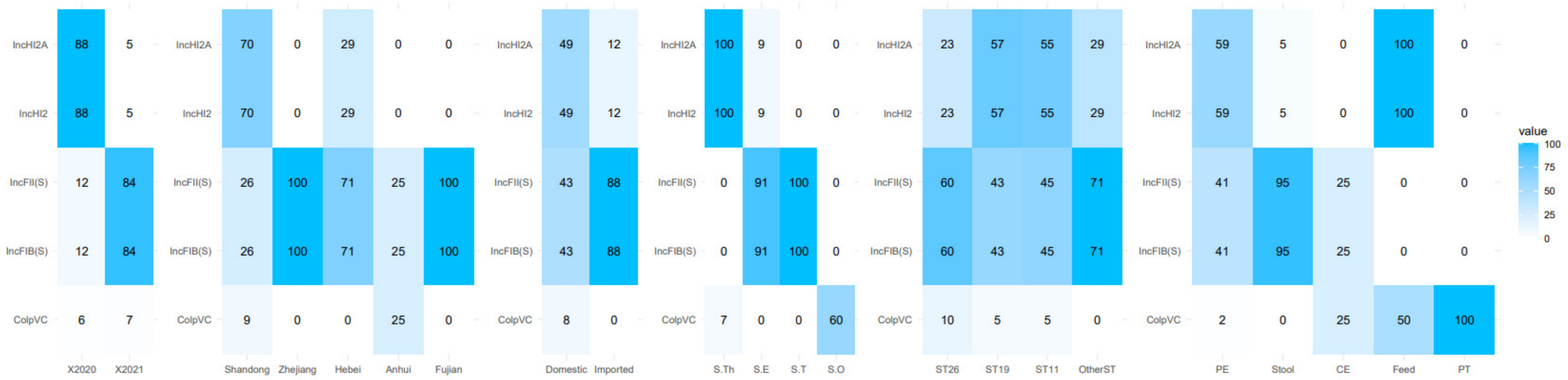

Figure S3. The heatmap of plasmids distribution in *Salmonella* isolates.

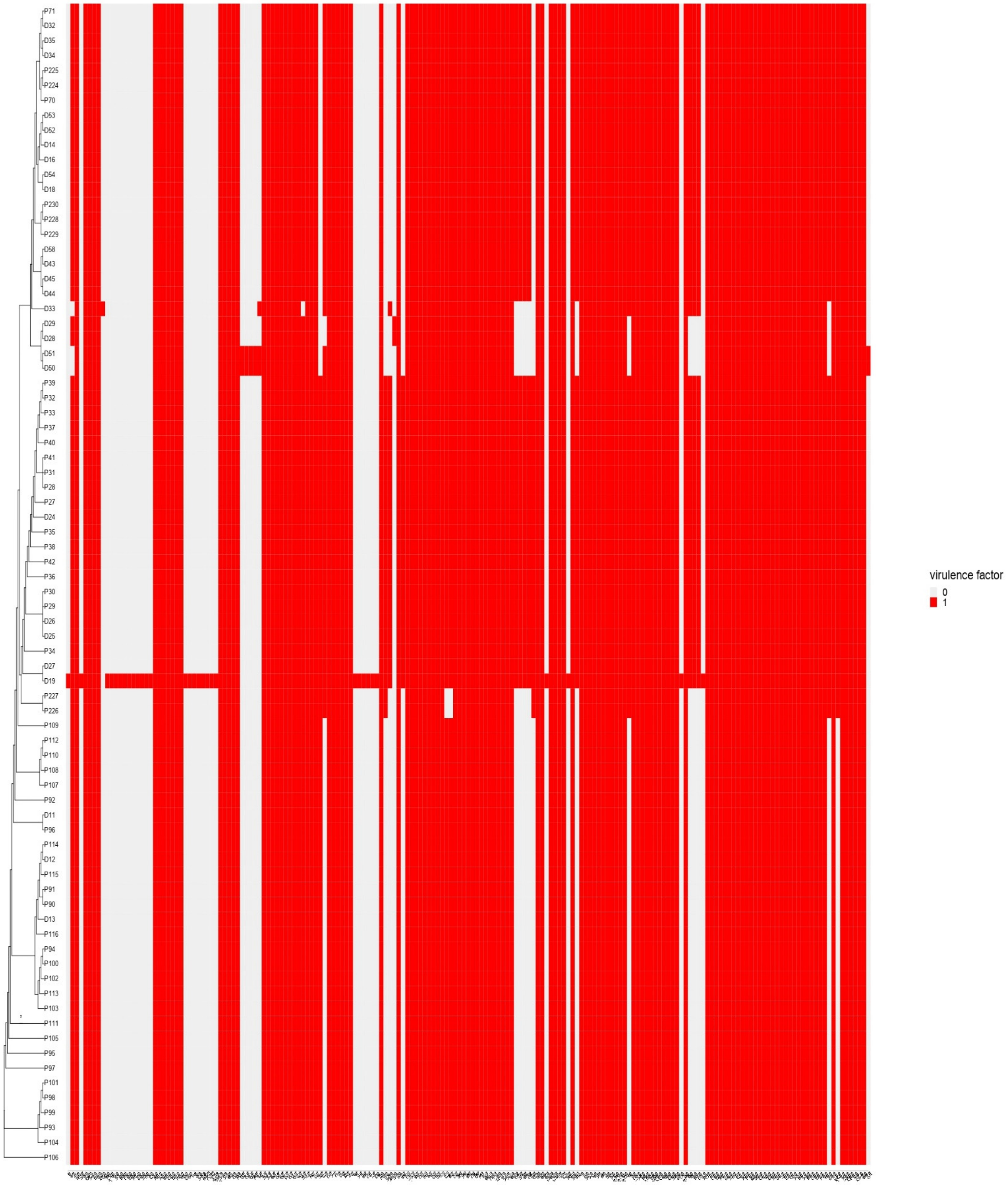

Figure S4. Virulence gene detection based on WGS of *Salmonella* strains.
